# Complete plastome sequence of *Vatica mangachapoi* (Dipterocarpaceae): a vulnerable (VU) plant species in Southeast Asia

**DOI:** 10.1080/23802359.2018.1522977

**Published:** 2018-10-03

**Authors:** Jian-Hua Wang, Kun-Kun Zhao, Zhi-Xin Zhu, Hua-Feng Wang

**Affiliations:** Hainan Key Laboratory for Sustainable Utilization of Tropical Bioresources, Institute of Tropical Agriculture and Forestry, Hainan University, Haikou, China

**Keywords:** *Vatica mangachapoi*, plastome, phylogeny, genome structure, Dipterocarpaceae

## Abstract

*Vatica mangachapoi* is a tree up to 20 m tall with white resinous. It is distributed in China (Hainan province), Indonesia, Malaysia (N Borneo), Philippines, Thailand, and Vietnam. It grows in forests on hills and mountain slopes below 700 metres. Its durable wood is used for making boats and building bridges and houses. It has been ranked as a VU (Vulnerable) species in China. Here we report and characterize the complete plastid genome sequence of *V. mangachapoi* in an effort to provide genomic resources useful for promoting its conservation and phylogenetic research. The complete plastome is 151,538 bp in length and contains the typical structure and gene content of angiosperm plastome, including two Inverted Repeat (IR) regions of 23,921 bp, a Large Single-Copy (LSC) region of 83,587 bp and a Small Single-Copy (SSC) region of 20,109 bp. The plastome contains 114 genes, consisting of 80 unique protein-coding genes, 30 unique tRNA gene, and 4 unique rRNA genes. The overall A/T content in the plastome of *V. mangachapoi* is 62.80%. The phylogenetic analysis indicated that *V. mangachapoi* and *V. odorata* is closely related and as an independent branch in Malvales in our study. The complete plastome sequence of *V. mangachapoi* will provide a useful resource for the conservation genetics of this species and for the phylogenetic studies for *Vatica*.

*Vatica mangachapoi* Blanco (Dipterocarpaceae, Malvales) is a tree to 20 m tall with white resinous. It is distributed in China (Hainan province), Indonesia, Malaysia (N Borneo), Philippines, Thailand, and Vietnam. It grows in forests on hills and mountain slopes below 700 metres. Its durable wood is used for making boats and building bridges and houses (Li et al. 2007). It has been ranked as a VU (Vulnerable) species in China (Qin et al. 2017). Consequently, the genetic and genomic information is urgently needed to promote its systematics research and the development of conservation value of *V. mangachapoi*. Here, we report and characterize the complete plastome of *V. mangachapoi* (GenBank accession number: MH716496, this study). This is the first report of a complete plastome for *V. mangachapoi.*

In this study, *V. mangachapoi* was sampled from Diaoluo Mountain (18.67°N, 109.88°E), which is a National Nature Reserve of Hainan, China. A voucher specimen (Wang et al. B61) was deposited in the Herbarium of the Institute of Tropical Agriculture and Forestry (HUTB), Hainan University, Haikou, China.

The modified cetyltrimethyl ammonium bromide (CTAB) protocol of Doyle and Doyle (1987) was used to extract genomic DNA from dry leaf tissues. The genomic DNA of each sample was quantified and analyzed with Agilent 2100 BioAnalyzer (UCDAVIS Genome Center, Davis, California, USA). Samples yield at least 0.8 μg DNA were selected for subsequent libraries construction and *de novo* sequencing. Genomic DNA of selected samples was used to build the paired-end libraries with 200–400 bp insert size. Libraries were sequenced using BGISEQ-500 platform at BGI (Shenzhen, China) and produced about 8 Gb high quality per sample with 100 bp paired-end reads. Raw reads were trimmed using SOAPfilter_v2.2 (BGI-Shenzhen, China) with the following criteria (1) reads with >10% base of N; (2) reads with >40% of low quality (value ≤10); (3) reads contaminated by the adaptor and produced by PCR duplication. Around 6 Gb clean data were assembled against the plastome of *Vatica odorata* (KX966283.1) (Cvetković et al. 2017) using MITO bim v1.8 (Natural History Museum, University of Oslo, Oslo, Norway) (Hahn et al. 2013).

The plastome was annotated using Geneious R8.0.2 (Biomatters Ltd., Auckland, New Zealand) against the plastome of *Vatica odorata* (KX966283.1). The annotation was corrected with DOGMA (Wyman et al. 2004).

The plastome of *V. mangachapoi* was found to possess a total length 151,538 bp with the typical quadripartite structure of angiosperms, containing two Inverted Repeats (IRs) of 23,921 bp, a Large Single-Copy (LSC) region of 83,587 bp, and a Small Single-Copy (SSC) region of 20,109 bp. The plastome contains 114 genes, consisting of 80 unique protein-coding genes (seven of which are duplicated in the IR: *rps12, rps7, ndhB, ycf15, ycf2, rpl23,* and *rpl2*), 30 unique tRNA genes (seven of which are duplicated in the IR: *trnN-GUU, trnR-ACG, trnA-UGC, trnI-GAU, trnV-GAC, trnL-CAA,* and *trnI-CAU),* and 4 unique rRNA genes (5S rRNA, 4.5S rRNA, 23S rRNA, and 16S rRNA). Among these gene, 3 pseudogenes [*infA* (translation from 80,371 to 80,122), *ycf1* (translation from 107,676 to113,165), *ycf15* (translation from 92,974 to 93,165 and 142,151 to 141,960)], 12 genes (*trnK-UUU, trnL-UAA, trnV-UAC, trnI-GAU, trnA-UGC, rpl2, rpoC1, petB, petD, ndhB, ndhA, rps12*) possessed a single intron and a gene (*ycf3*) had two introns. The gene *rps12* was found to be trans-spliced, as is typical of angiosperms. The overall A/T content in the plastome of *V. mangachapoi* is 62.80%, which the corresponding value of the LSC, SSC, and IR region were 64.80%, 68.70%, and 56.90%, respectively.

We used RAxML (Stamatakis 2006) with 1000 bootstraps under the GTRGAMMAI substitution model to reconstruct a maximum likelihood (ML) phylogeny of 11 published complete plastomes of Malvales, using *Bretschneidera sinensis* (Akaniaceae, Brassicales) as an outgroup. The phylogenetic analysis indicated that *V. mangachapoi* and *V. odorata* is closely related and as an independent branch in Malvales in our study (Figure 1). Most nodes in the plastome ML trees were strongly supported. The complete plastome sequence of *V. mangachapoi* will provide a useful resource for the conservation genetics of this species, as well as for the phylogenetic studies for *Vatica*.

**Figure 1.**
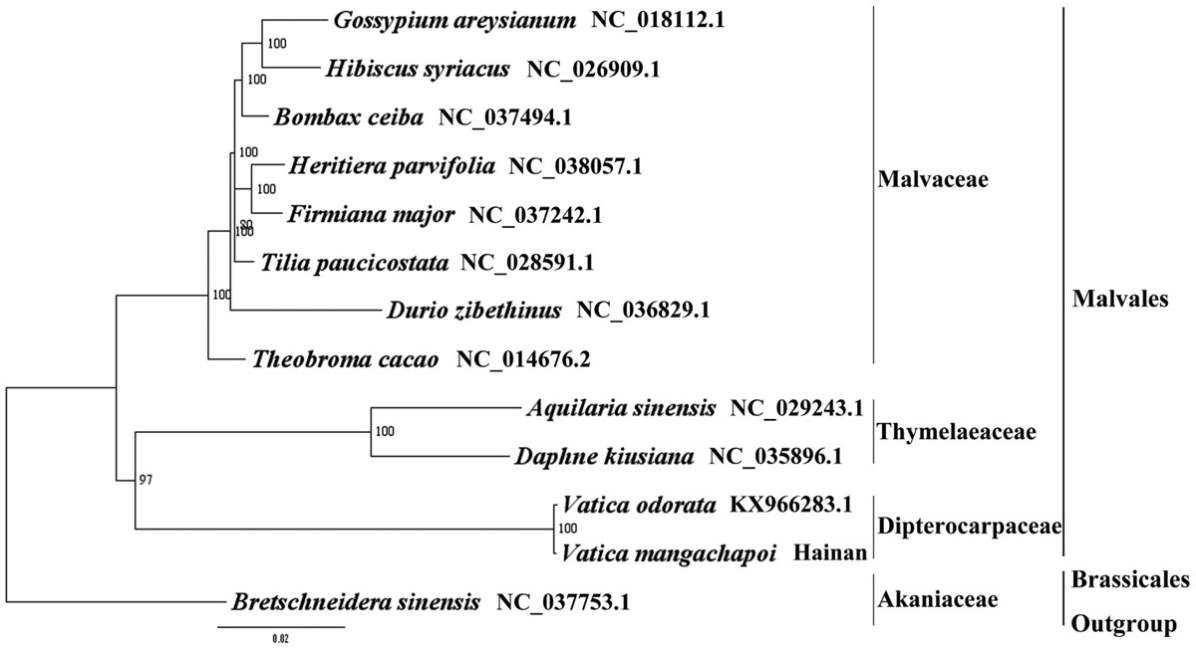
The best ML phylogeny recovered from the combined sequences of the 75 coding genes by RAxML. Accession number: *Vatica mangachapoi* (Genbank accession number: MH716496, this study)*, Vatica odorata* KX966283.1, *Daphne kiusiana* NC_035896.1*, Aquilaria sinensis* NC_029243.1*, Theobroma cacao* NC_014676.2, *Durio zibethinus* NC_036829.1*, Tilia paucicostata* NC_028591.1, *Firmiana major* NC_037242.1*, Heritiera parvifolia* NC_038057.1*, Bombax ceiba* NC_037494.1*, Hibiscus syriacus* NC_026909.1, *Gossypium areysianum* NC_018112.1, outgroup: *Bretschneidera sinensis* NC_037753.1.
